# A MD Simulation and Analysis for Aggregation Behaviors of Nanoscale Zero-Valent Iron Particles in Water via MS

**DOI:** 10.1155/2014/768780

**Published:** 2014-08-27

**Authors:** Ying Zhao, Dongmei Liu, Huan Tang, Jing Lu, Fuyi Cui

**Affiliations:** ^1^School of Municipal and Environment Engineering, Harbin Institute of Technology, Harbin, Heilongjiang 150090, China; ^2^State Key Laboratory of Urban Water Resource and Environment, Harbin Institute of Technology, Harbin 150090, China

## Abstract

With the development of nanotechnology, more nanomaterials will enter into water environment system. Studying the existing form of nanomaterials in water environment will help people benefit from the correct use of them and to reduce the harm to human caused by them for some nanomaterials can bring polluting effect. Aggregation is a main behavior for nanoparticle in water environment. NZVI are used widely in many fields resulting in more NZVI in water environment. Molecular dynamics simulations and Materials Studio software are used to investigate the microaggregation behaviors of NZVI particles. Two scenes are involved: (1) particle size of NZVI in each simulation system is the same, but initial distance of two NZVI particles is different; (2) initial distance of two NZVI particles in each simulation system is the same, but particle size of NZVI is different. Atomistic trajectory, NP activity, total energy, and adsorption of H_2_O are analyzed with MS. The method provides new quantitative insight into the structure, energy, and dynamics of the aggregation behaviors of NZVI particles in water. It is necessary to understand microchange of NPs in water because it can provide theoretical research that is used to reduce polluting effect of NPs on water environment.

## 1. Introduction

NPs are ultrafine particles with lengths between 1 nm and 100 nm in two or three dimensions [1, 2]. The nanomaterials made from NPs have novel physical and chemical properties, such as unique optical, electronic, or mechanical properties, which other materials do not have. So it makes nanomaterials to be widely used in many fields, such as energy, coatings, cosmetics, medicine, textile, and electronics. It is predicted that more and more nanomaterials will enter into the market in future [3, 4]. Vast NPs will get into water environment obviously for the applications of nanomaterials.

It shows that many nanomaterials can bring polluting effect on water environment and hurt humans for specific and unique shape, quantum size effect, surface effect, macroquantum tunnel effect, bioactivity, or potential toxicity [5–14]. For vast application of nanotechnology and potential environmental influence of nanomaterials, studying the existing form of nanomaterials in water environment will help people benefit from the correct use of them and to reduce the harm to human caused by them.

It is indicated that NPs possess surface effect. The aggregation is easy to occur when two NZVI particles approach. So aggregation is a main behavior for NPs in water environment. NPs are between 1 and 100 nanometers in size in production, but they usually gather colloidal substance with bigger particle size in water environment [15]. Stability of NPs depends on the intermolecular forces among them, such as van der Waals force and Coulomb force. Intermolecular forces of NPs are influenced by the size of NPs and their distance, so which can affect the aggregation behavior of NPs in water.

Now the methods that are widely used to measure NPs in medium are hardly quantitatively monitor surface microcharacteristic and dynamic change of NPs. Molecular dynamics (MD) is a computer simulation of physical movements of atoms and molecules in the context of N-body simulation. The atoms and molecules are allowed to interact for a period of time, giving a view of the motion of the atoms. In the most common version, the trajectories of atoms and molecules are determined by numerically solving Newton's equations of motion for a system of interacting particles, where forces between the particles and potential energy are defined by molecular mechanics force fields. The method was originally conceived within theoretical physics in the late 1950s [16, 17] but is applied today mostly in chemical physics, materials science, and the modeling of biomolecules. Now MD is an indispensable method to research microcharacteristics of NPs in atomic or molecular scale [18].

The research of microcharacteristics of NPs with MD is greatly concerned by many scholars, including surface properties, microstructure, and mechanism of action of NPs in interface, gas, and liquid. Levchenko et al. [19] uses MD simulations in combination with embedded atom method potential to study the alloying reaction in an Al-coated Ni NP with equiatomic fractions and a diameter of ~4.5 nm. Rudyak et al. deal with a MD simulation of the diffusion of NP in dense gases and liquids using the Rudyak-Krasnolutskii NP-molecule potential [20, 21]. Relations are obtained between the diffusion coefficient of NP and the NP radius and the temperature of the medium. Zhang et al. [22] uses MD to study the response of NP structure to different types of surface environments. Frost and Dai [23] have studied the self-assembly of hydrophobic NP at ionic-liquid- (IL-) water and IL-oil (hexane) interfaces using MD simulations. The charge of NP surface and changes of charge density distribution in interfaces are calculated.

MD simulation is seldom used to study the microchange of NP in water. The existing researches mainly focus on polarity of natural organic matter (NOM) in water and complexation between NOM and metallic ion. Some researchers [24, 25] investigate the interactions of metal cations with the carboxylic groups of a model NOM fragment and acetate anions in aqueous solutions by computational MD. The stabilizing effect of water molecule bridges on polar regions in humic substances has been investigated with MD simulations by Aquino et al. [26].

MD simulations and MS software are used to study the microaggregation process of NZVI particles in water. The constraints are needed to set up several amorphous cells (simulation systems), including changes of distance between two NZVI particles or particle size of the NZVI. The aggregation behaviors of NZVI particles are computed quantitatively in atomic or molecular scale. Atomistic trajectory, NP activity, total energy, and adsorption of H_2_O are analyzed with MS. The methods provide new quantitative insight into the structure, energy, and dynamics of the aggregation behaviors of NZVI particles in water. It is necessary to understand microchange of NPs in water because it can provide theoretical research used to reduce polluting effect of NPs on water environment.

## 2. Materials and Methods

### 2.1. NZVI and MD Simulation

#### 2.1.1. NZVI

NZVI particles are Fe^0^ NP between 1 and 100 nanometers in size. NZVI is widely used in many fields [27–29]. Furthermore, NZVI is a good material to remove difficult degradation pollutant from wastewater and restore the polluted ground water [30–32]. The excellent properties make NZVI used widely resulting in more NZVI in water environment. The research of aggregation behaviors of NZVI particles in water is significant for water quality safety.

#### 2.1.2. MS and the Application

Materials Studio (MS) is used in the paper to address MD simulation. MS dramatically increases the accessibility of computational materials science. It delivers powerful methods to chemists, materials scientist, and engineers in a flexible and easy-to-use environment. It makes it straightforward to communicate ideas related to materials structure and properties and to solve critical problems in the chemicals and materials industries.

Structure optimizations of NZVI particle and water molecule are done with MS. Forcite. It can search for a minimum energy structure. The Forcite Geometry Optimization task allows you to refine the geometry of a structure until it satisfies certain specified criteria. This is done using an iterative process, in which the atomic coordinates, and possibly the cell parameters, are adjusted until the total energy of the structure is minimized. In general, therefore, the optimized structure corresponds to a minimum in the potential energy surface.

The simulation systems are set up with MS. amorphous cell. Amorphous cell is a suite of computational tools that allow you to construct representative models of complex amorphous systems and to predict key properties. Among the properties that you can predict and investigate are cohesive energy density, equation-of-state behavior, chain packing, and localized chain motions. The methodology of amorphous cell construction is based on an extension of well-established methods for generating bulk disordered systems containing chain molecules in realistic equilibrium conformations.

MS. Discover is used to implement MD simulations. Discover offers powerful atomistic simulation methods that can be applied to a wide range of molecules and materials. Discover is Materials Studio's “simulation engine.” It incorporates a broad spectrum of molecular mechanics and dynamics methodologies that have demonstrated applicability to molecular design.

The Compass Force field is used in the whole simulations. The Compass Force field has broad coverage in covalent molecules including most common organics, small inorganic molecules, and polymers. The latest development in COMPASS extended the coverage to include inorganic materials: metals, metal oxides, and metal halides using various noncovalent models. Some of these materials have been parameterized [33].

### 2.2. The Aggregation Behavior of NZVI Particles

#### 2.2.1. MD Simulation Design

The objective of the simulation is to observe the aggregation effect and trajectory of NZVI particles in water in different condition. There are two scenes based on different conditions: (1) particle size of NZVI in each simulation system is the same, but initial distance of two NZVI particles is different; (2) initial distance of two NZVI particles in each simulation system is the same, but particle size of NZVI is different. Considering above scenes, the simulations are, respectively, named simulation (1) and simulation (2). There are two NZVI particles and many water molecules in all simulation system.

The particle size of NZVI in simulation (1) is 1 nm. The amorphous cell (simulation system) is an irregular cell, the distance of two NZVI particles is formed randomly. Five simulation systems are selected as study objects according to the different distance from small to large in simulation (1). There are two simulation systems in simulation (2). The particle size is also 1 nm in the first one simulation system, but it is 1.5 nm in the other one. The initial distances of two NZVI particles are the same in their minimization systems, which are the initial conformations for simulation.

The initial distance of two NZVI particles and particle size of NZVI should influence the aggregation behavior, so influencing differences are analyzed with MS, including atomistic trajectory, NP activity, total energy, and adsorption of H_2_O.

#### 2.2.2. Molecular Optimization and Simulation System

**Figure 1:**
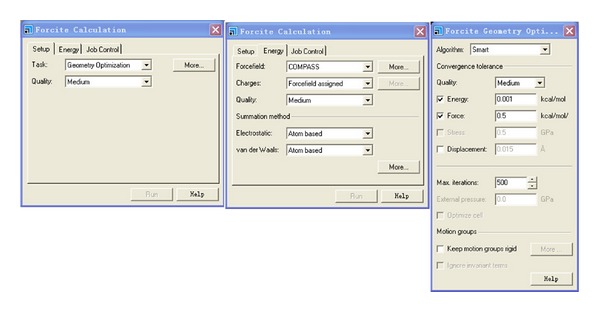
The details of the simulation parameters for structure optimizations of NZVI particle and water molecule.

Water molecule and NZVI particle with two sizes are optimized by MS. Forcite as basic elements to set up initial simulation system. The details of the simulation parameters are shown in Figure 1 for structure optimizations of NZVI particle and water molecule.

**Figure 2:**
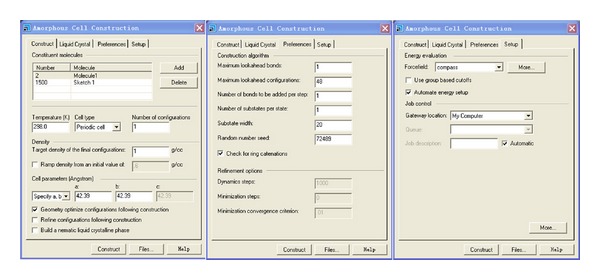
The details of the simulation parameters for setting up the simulation systems.

The simulation system is a periodic cell. For making MD simulation work reasonably and reducing the calculation amount, the number of water molecule in simulation system should be moderate. From many tests of system construction, it is determined that there are 1500 water molecules in all simulation systems. The density of initial system is 1 g/c^3^. The details of the simulation parameters are shown in Figure 2 for setting up the simulation systems.

#### 2.2.3. Minimization System

**Figure 3:**
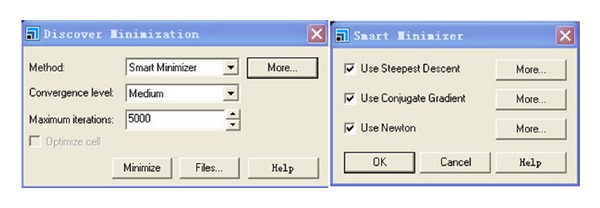
The details of the simulation parameters for minimization.

Typically, after a structure is built, it must be refined to produce a stable conformation. This refinement process is known as minimization (or geometry optimization). Minimization is an iterative procedure in which the coordinates of the atoms and possibly the cell parameters are adjusted so that the total energy of the structure is reduced to a minimum (on the potential energy surface). Minimization results in a structural model which closely resembles the experimentally observed structure [33]. Furthermore, the simulation system is an irregular cell and molecules do not distribute uniformly in it. So there are many empty spaces in system. Minimization can remove the empty spaces and make the molecules distribute uniformly, which can make simulation results more accurate. Minimization is necessary in MD simulation. Minimization is done with MS. Discover (Figure 3).

#### 2.2.4. MD Simulation

**Figure 4:**
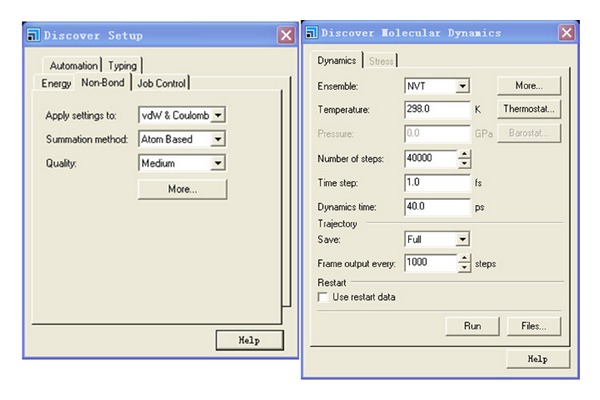
The details of the simulation parameters of system 1 for MD simulation.

The MD simulation is implemented with MS. Discover. All parameters are designed according to simulation conditions, including force field type, nonbond tab, thermodynamic ensemble, target temperature, and dynamics time. Force field type is a key parameter in simulation. It is necessary to define force field type for each kind of atom in system. The details of the simulation parameters of system 1 are shown in Figure 4 for MD simulation.

#### 2.2.5. Analytical Method of MD Simulation

(*1) Trajectory of NZVI Particle*. Dynamics allows you to save the results of a dynamics run as a trajectory file, which is essentially a series of snapshots of the simulation taken at regular intervals. Trajectories can include data such as coordinate, model structure, minimized-model structure, temperature, energies, volume, pressure, cell parameters, and stress. The information is useful for MD research. In this study, we use trajectory files for computing the distance of two NZVI particle and building the relationship between the distance and time. The distance at any specified time can be observed and it helps us to analyze the whole aggregation process.

(*2) Total Energy Analysis*. Molecules and atoms of the simulation systems move all the time. They can be attracted, repelled, and collided in the movement. So energy would be produced in the system. Total energy of the system changes with time and it can be calculated with MS. The change of total energy is related to the distance of two NZVI particles. The correlation analysis of the total energy and distance is done.

(*3) Mean Square Displacement Analysis*. The most prominent performance of MD simulation is to calculate the dynamic characteristic of the molecules and atoms in system. Mean square displacement (MSD) is introduced to investigate the change of NZVI particle activity in whole simulation process. Atoms of a simulation system move all the time and their positions are not the same at different moment. Supposing *r*_*i*_(*t*) is the position of atom *i* at moment *t*, the MSD of atoms in a simulation can be computed by its definition

(1) $$\begin{array}{c} \text{MSD}=R\left(t\right)=\langle {\left|r(t)-r(0)\right|}^{2}\rangle , \end{array}$$

where 〈〉 denotes averaging over all the atoms (or all the atoms in a given subclass).

It is inferred that the slope of some point in MSD curve is directly proportional to displacement increment of this point from initial position in very short time. To some extent, displacement is related to NZVI particle activity. The big displacement increment indicates stronger activity. So we can get the change of NZVI particle activity in simulation process by observing the variation of MSD curve slope. It is useful to correlate distance of two NZVI particles and their activity.

(*4) Radial Distribution Function Analysis*. Radial distribution function (RDF) in a system of particles (atoms, molecules, colloids, etc.) describes how density varies as a function of distance from a reference particle. The RDF gives a measure of the probability that, given the presence of an atom at the origin of an arbitrary reference frame, there will be an atom with its center located in a spherical shell of infinitesimal thickness at a distance, *r*, from the reference atom. This concept also embraces the idea that the atom at the origin and the atom at distance *r* may be of different chemical types, say *α* and *β* [33]. The resulting function is then commonly given the symbol *g*_*αβ*_(*r*). Here MSD is used to investigate the changed density of water molecules around NZVI particle in simulation system. It is useful to analyze the adsorption characteristic of water molecules around NVZI particle in whole simulation process.

## 3. Results and Discussion

### 3.1. Simulation of Distance Influence (Simulation (1))

#### 3.1.1. Conformations of Initial and Minimization System

**Figure 5:**
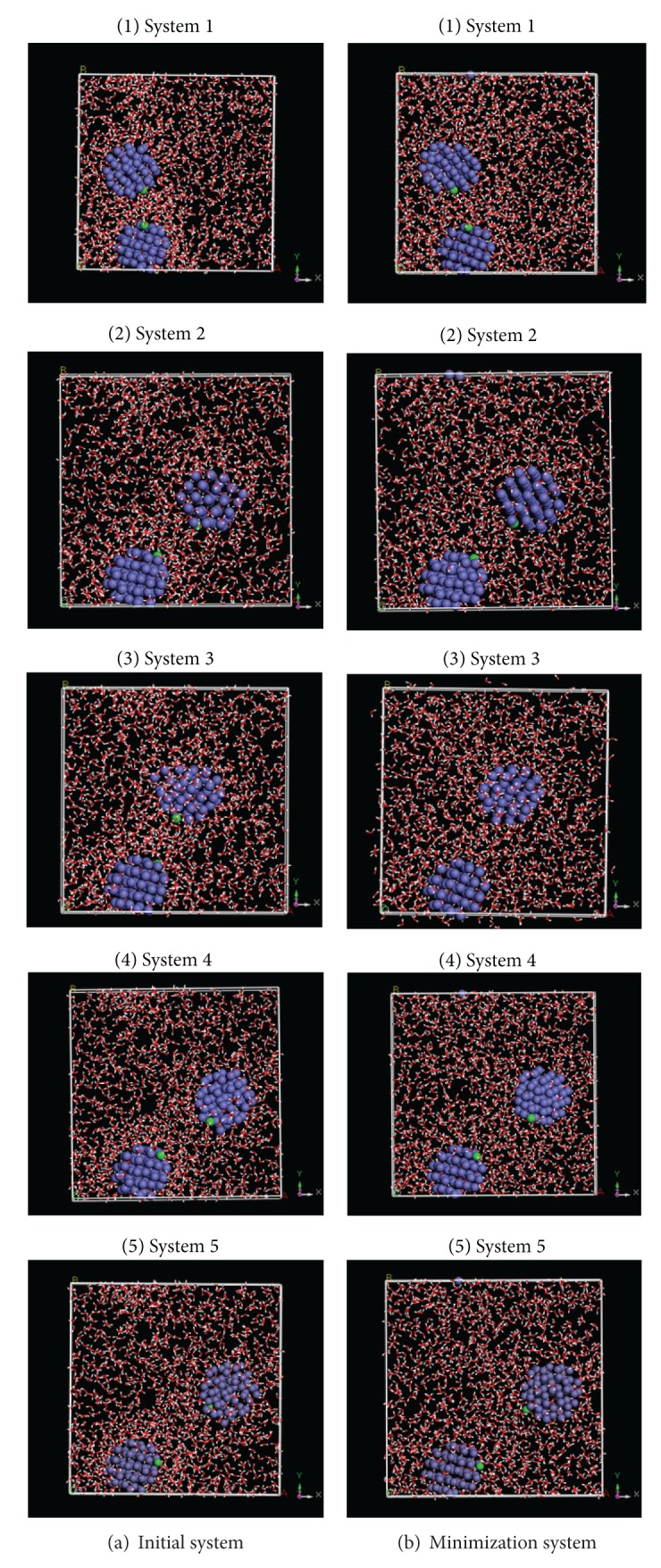
Conformations of initial system and minimization system of systems 1–5.

The changed initial distance of two NZVI particles should influence the aggregation behavior. According to above design of MD simulation (1), there are two NZVI particles and 1500 water molecules in simulation system. The particle size of NZVI is 1 nm. A group of simulation systems are setup. Five systems are selected from them based on initial distance of 2 NZVI particles, which is from small to large (Table 1). Five systems are named, respectively, systems 1–5. The conformations of initial system (a) and minimization system (b) are shown in Figure 5.

Contrasting with initial systems, the distances of two NZVI particles in minimization systems increase slightly. The distances of initial system and minimization system are shown in Table 1.

The conformations of initial system reveal that the water molecules adsorb on the NZVI particles so that molecules do not distribute uniformly and there are many empty spaces in initial system. On the other hand, minimization can remove the empty spaces and make the molecules distribute uniformly, which can be seen in Figure 5(b).

#### 3.1.2. Trajectory Analysis of NZVI Particle

**Figure 6:**
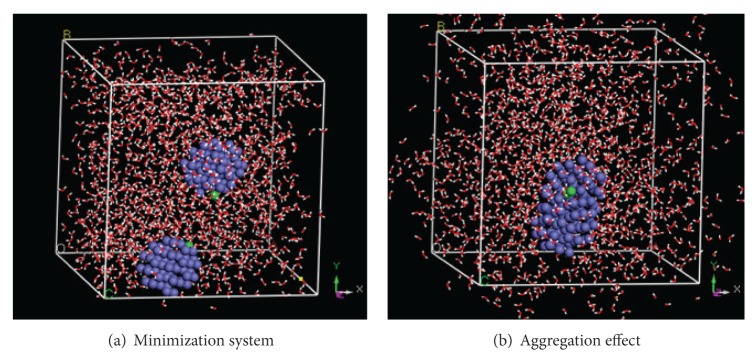
Conformations of minimization system and aggregation effect of system 3.

The conformation of minimization system is the beginning of MD simulation. The appropriate parameter values and iterations are defined for five simulations. The simulations indicate that two NZVI particles can aggregate in all systems. The aggregation time is defined as the time from beginning to original contact of two NZVI particles. The aggregation time is related to the initial distance of two NZVI particles. The aggregation effect of system 3 is shown as an example in Figure 6(b).

**Figure 7:**
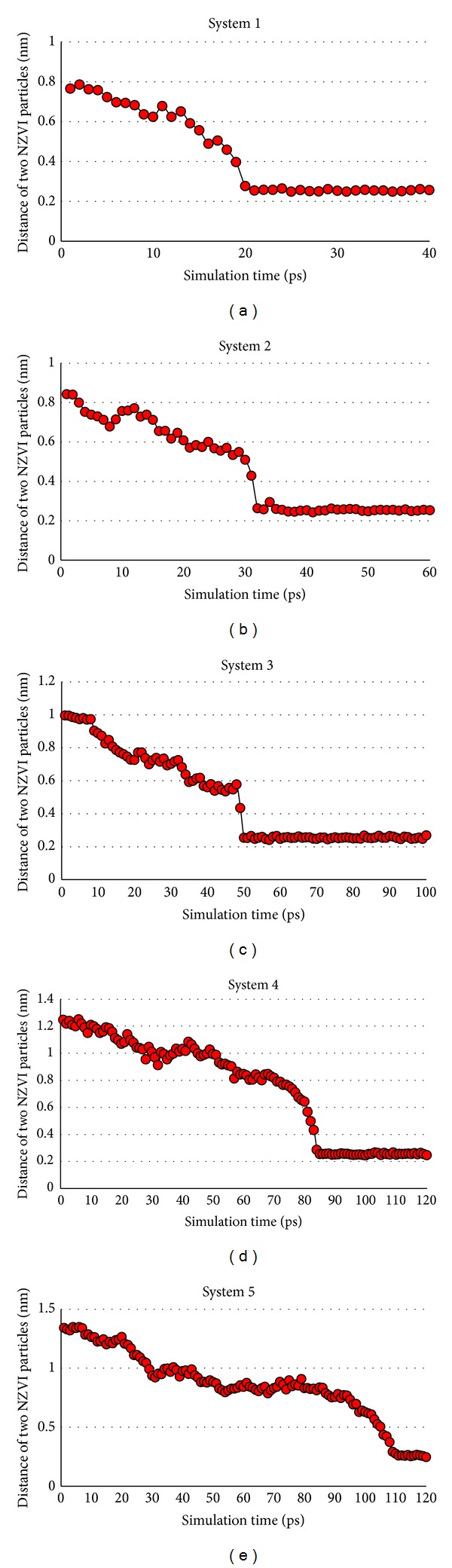
Distance change of two NZVI particles in whole simulation process of systems 1–5.

It is difficult to express the distance of two NZVI particles by standard mathematical formula as the shape of NZVI particle is not regular. In this study two atoms in near surface of two NZVI particles are selected, which are named characteristic atoms, to express the distance of two NZVI particles. It is a fact that the characteristic atoms can rotate so that the distance of them is not drop all the time, as can be seen in Figures 7 and 13. Considering above fact, many pairs of characteristic atoms in near surface of two NZVI particles are tested, respectively. On the whole, the rotational effects of characteristic atoms influence the aggregation time slightly from the conclusion of the tests. We find that the two atoms, which contact first in aggregation process, are always with the closer initial distance. So the characteristic atoms are selected based on two requirements. Firstly, the two atoms are from near surface of two NZVI particles. Secondly, the atoms contact first in the aggregation process. The trajectories of characteristic atoms (green Fe atoms in Figure 6) are collected and taken as distances of two NZVI particles (Figure 7).

Internal structure of NZVI particle is studied and it indicates minimum distance of atoms in NZVI is about 0.25 nm. The minimum distance is the distance between the centers of two atoms when there is one atom next to another one. So it reveals that two NZVI particles begin to aggregate when their distance is about 0.25 nm. In general, there are three stages in whole simulation process. Brownian motion is the random motion of particles suspended in a fluid resulting from their collision with quick atoms or molecules in liquid [34]. Firstly, the force between two NZVI particles is weak because their distance is not so close. The Brownian motion of NZVI particles and water molecules always exist in the simulation system. Secondly, the two NZVI particles approach each other in one time by the continuous Brownian motion. The force between NZVI particles increases as their distance is reduced. At this time the van der Waals force and Coulomb force become main factors to influence the distance of two NZVI particles. Thirdly, when the distance is very small and reaches some value (defined as critical point), the two NZVI particles move quickly because metallic bonds will be formed in the atoms from near surface of two NZVI particles. The powerful attractive forces from metallic bonds make the atoms aggregate quickly and soon new aggregation of NZVI particle reaches a stable state. It is indicated that NPs possess surface effect. Metallic bonds are easy to form in the atoms from near surface of two NZVI particles when they approach. They want to be more stable structure.

There are two characteristics in simulation process. Firstly, no matter how the initial distance changes, they approach more quickly when the distances reach some value (defined as critical point). The critical points of five simulations are similar and they are about 0.5 nm. Secondly, aggregation time is related to initial distance of two NZVI particles. The average aggregation speed is defined as *v*:

(2) $$\begin{array}{c} v=\frac{D}{T}, \end{array}$$

where *D* is displacement distance of two NZVI particles and *T* is aggregation time. *D* is defined as

(3) $$\begin{array}{c} D=I-M, \end{array}$$

where *I* is initial distance in minimization system and *M* is minimum distance (0.25 nm) of Fe atoms in NZVI particle. The average aggregation speeds of five systems are shown in Table 2. It indicates that the average aggregation speed drops exponentially as the distance increases. Two NZVI particles with close range are easy to aggregate and they aggregate slowly when the distance increases obviously.

#### 3.1.3. Total Energy Analysis

**Figure 8:**
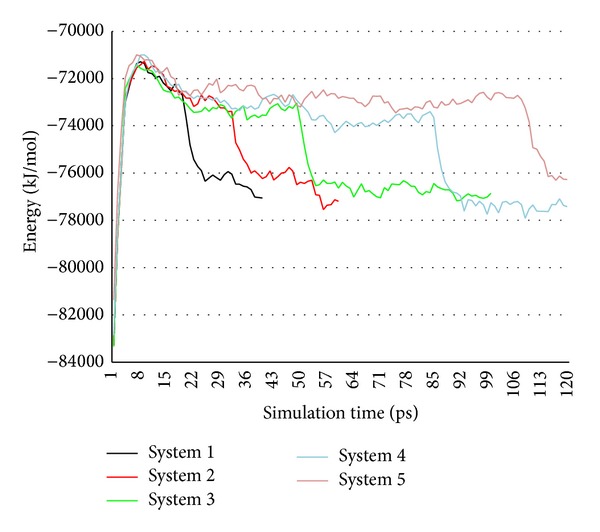
Curves of total energy in systems 1–5.

Curves of total energy in five systems are shown in Figure 8.

In general, there are four stages in whole simulation process from energy curves. Firstly, energies rise to the peak quickly at the beginning. Secondly, energies drop slowly before the aggregation. Thirdly, energies drop greatly when two NZVI particles aggregate. Finally, energies are also steady when new aggregation of NZVI particle reaches a stable state.

Though initial distance of two NZVI particles is different, initial total energies of five systems are similar. It indicates that initial total energy is only related to type and number of the molecules (atoms) in simulation system and is not related to the initial distance of the molecules (atoms). But the energy change is closely related to the trajectory of NZVI particle, which drop more quickly when two NZVI particles aggregate. The total energy is steady after the aggregation and the stable energies of five systems are similar. It indicates that the final energy of stable state is only related to final conformation and is not simulation process.

#### 3.1.4. MSD Analysis

**Figure 9:**
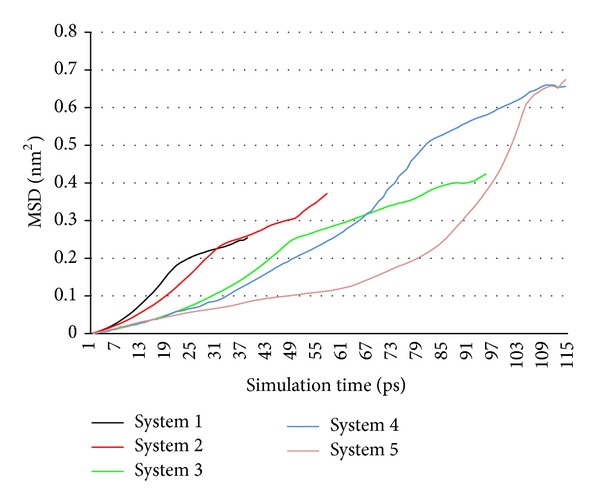
MSD curves of whole Fe atoms in systems 1–5.

Whole Fe atoms in the system are considered and MSD curves are shown in Figure 9.

The NZVI particle activity is related to the slope of some point in MSD curve (see Section 2.2.5(3)). Figure 9 indicates similar change rules in five systems. At the beginning of simulation, NZVI particle activity is low and it increases slowly. Next, the activity enhances obviously when the distance of two NZVI particles reaches the critical point. Finally, the activity drops again after the aggregation and it becomes steady gradually. The activities of five systems are similar after the aggregation. Contrasting five curves in Figure 9, it indicates that the activity has a greater fluctuation with the distance of two NZVI particles increasing. For example in system 5, the activity is lower than other systems at the beginning, and it increases greatly when the distance reaches the critical point.

#### 3.1.5. RDF Analysis

**Figure 10:**
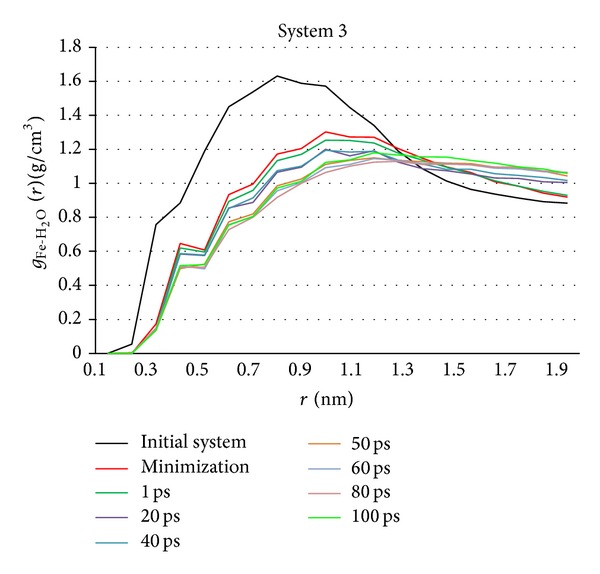
RDF curves of Fe-H_2_O of system 3 in key time.

Fe atoms and water molecules are considered, respectively, as two sets. RDF curves of Fe-H_2_O of system 3 are shown as an example in Figure 10. Nine key time are selected as the observed time. Figure 10 reveals changed density of water molecules around NZVI particle in whole simulation process. The density is the highest in initial system. It indicates that many water molecules adsorb on NZVI particle in initial system, which is consistent with Figure 5(a)(3). The density drops obviously after minimization as shown in Figure 5(b)(3). Next, there are two small declines in density in remaining time. One occurs at initial stage of the simulation and another one occurs after the aggregation. The density of other time is almost invariable.

### 3.2. Simulation of Particle Size Influence (Simulation (2))

#### 3.2.1. Conformation of Initial and Minimization System

**Figure 11:**
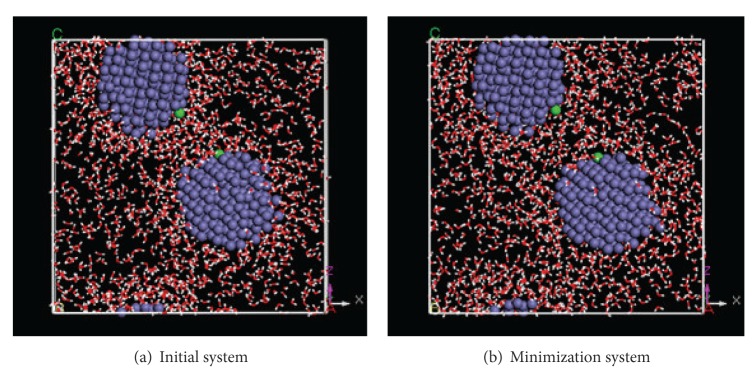
Conformations of initial and minimization system of system 6.

The changed particle size should influence the aggregation behavior of NZVI particles. According to the design of MD simulation (2), there are also two NZVI particles and 1500 water molecules in simulation system. System 3 is selected as one object. Another object is named system 6 and particle size of NZVI is 1.5 nm. Initial distances of two NZVI particles in two objects are the same in their minimization system.  Figure 11 is conformations of initial and minimization system of system 6. Contrasting Figure 11(a) and Figure 5(a)(3) indicates adsorption phenomenon of water molecules is more obvious when the particle size increases.

#### 3.2.2. Trajectory Analysis of NZVI Particle

**Figure 12:**
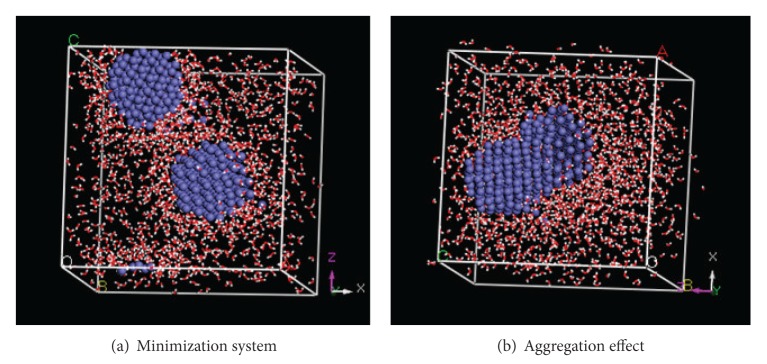
The conformations of minimization and aggregation effect of system 6.

**Figure 13:**
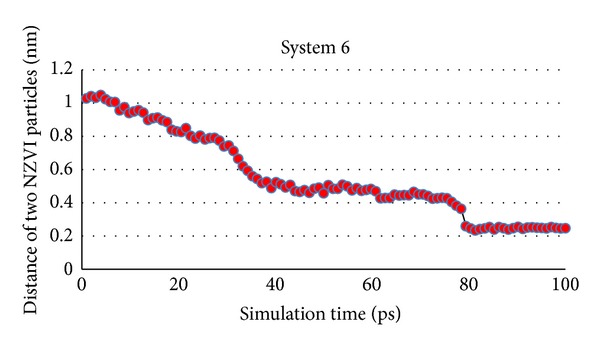
Distance change of two NZVI particles in whole simulation process of system 6.

Initial distance of two NZVI particles is 1 nm in system 6, which is the same as system 3. The simulation indicates that two NZVI particles can aggregate in system 6 (Figure 12(b)). The same method as simulation (1) is used to express the distance of two NZVI particles in simulation (2). Distance change of system 6 is shown in Figure 13.

Figure 13 reveals that there are also three stages in whole simulation process. Firstly, the force between two NZVI particles is weak because the distance of them is not so close. Secondly, the force between NZVI particles increases as their distance is reduced. At this time the van der Waals force and Coulomb force become main factors to influence the distance of two NZVI particles. Thirdly, when the distance is very small and reaches some value (defined as critical point), the powerful attractive forces from metallic bonds make the atoms aggregate quickly and soon new aggregation of NZVI particle reaches a stable state.

Although initial distance of two NZVI particles is the same in two systems, aggregation time with big particle size is longer than small size. It indicates that aggregation speed of small size is 1.58 times of big size. Furthermore, critical point for rapid contact is 0.4 nm in system 6, which is smaller than system 3 (0.5 nm). It should relate to the forces of systems. The repulsive force and repulsive force all increase when the particle size is bigger. But the increase of repulsive force is bigger than attractive force resulting in smaller critical point and difficult aggregation.

#### 3.2.3. Total Energy Analysis

**Figure 14:**
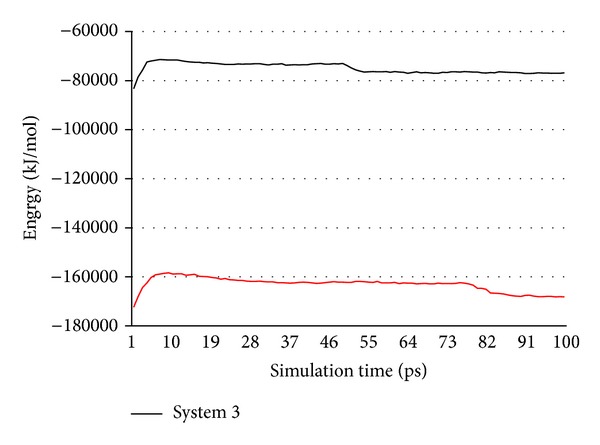
Curves of total energy in systems 3 and 6.

Total energy curves of systems 3 and 6 are shown in Figure 14. There are also four stages for energy change in whole simulation process of system 6, which is similar to system 3 (see Section 3.1.3). However, total energy enhances greatly as the particle size increases. Total energy of system 6 is more than 2 times of that of system 3.

#### 3.2.4. MSD Analysis

**Figure 15:**
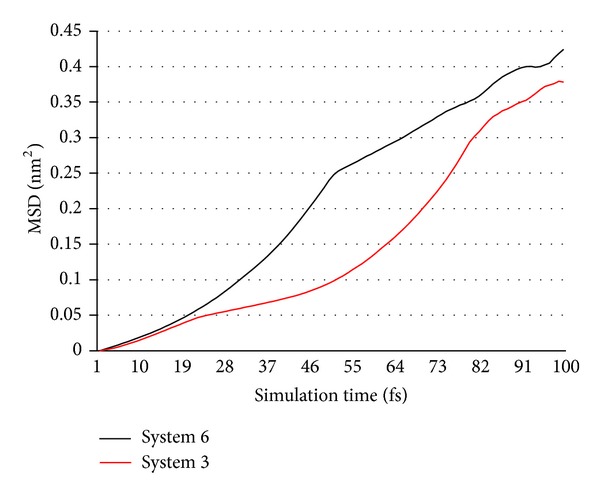
MSD curves of whole Fe atoms in systems 3 and 6.

Figure 15 indicates that the change rule of NZVI particle activity in system 6 is similar to that of system 3 (see Section 3.1.4). Furthermore, the activity has a greater fluctuation with the particle size increasing. The activity of system 6 is lower than system 3 at the beginning, and it increases greatly when the distance reaches the critical point.

#### 3.2.5. RDF Analysis

**Figure 16:**
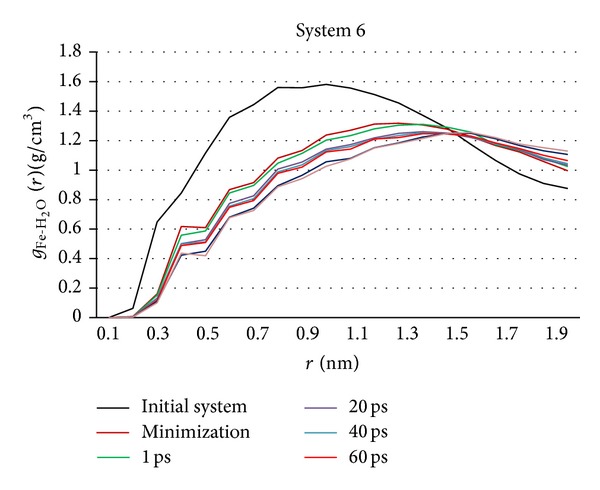
RDF curves of Fe-H_2_O of system 6 in key time.

RDF curves of system 6 (Figure 16) are calculated with the same method as system 3. There is similar change rule of density in system 6 and system 3 (see Section 3.1.5). However, water molecules adsorbing on NZVI particle in system 6 are thicker than that of system 3.

## 4. Conclusions

(1) The minimization system is the beginning of MD simulation. The simulations indicate that two NZVI particles can aggregate in six systems when their distance and size particle change. There are three stages in whole simulation process. Firstly, the force between two NZVI particles is weak because their distance is not so close. Secondly, the force between NZVI particles increases as their distance is reduced. At this time the van der Waals force and Coulomb force become main factors to influence the distance of two NZVI particles. Thirdly, when the distance reaches critical point, the powerful attractive forces from metallic bonds make the atoms aggregate quickly and soon new aggregation of NZVI particle reaches a stable state. Average aggregation speed drops exponentially with the distance increasing when NZVI particle size is the same. When NZVI particle size increases from 1 nm to 1.5 nm, aggregation speed of small size is 1.58 times that of big size, and critical point for rapid contact is smaller. It should relate to the forces of systems. When particle size increases, the increase of repulsive force is bigger than attractive force resulting in smaller critical point and difficult aggregation.

(2) In general, there are four stages for energy change in whole simulation process of six systems. Firstly, energies rise to the peak quickly at the beginning. Secondly, energies drop slowly before the aggregation. Thirdly, energies drop greatly when two NZVI particles aggregate. Finally, energies are also steady when new aggregation of NZVI particle reaches a stable state. It indicates that initial total energy is only related to type and number of the molecule (atom) in system and is not the initial distance of them. But the energy change is closely related to the trajectory of NZVI particle, which drops more quickly when two NZVI particles aggregate. The final energy of stable state is only related to final conformation and is not simulation process. The total energy enhances greatly as the particle size increases.

(3) There is similar change rule of NZVI particle activity in six systems. At the beginning of simulation, NZVI particle activity is low and it increases slowly. Next, the activity enhances obviously when the distance of two NZVI particles reaches the critical point. Finally, the activity drops again after the aggregation and it becomes steady gradually. It indicates that the activity has a greater fluctuation as the distance of two NZVI particles increases. The activity with bigger distance is lower than other systems at the beginning, and it increases greatly when the distance reaches the critical point. Similarly, the activity has a greater fluctuation as the particle size increases.

(4) Water molecules density around NZVI particle changes in the similar rules in six systems. The density is the highest in initial system. It indicates that many water molecules adsorb on NZVI particle in initial system. The density drops obviously after minimization. Next, there are two small declines in density in remaining time. One occurs at initial stage of the simulation. The other occurs after the aggregation. The density of other time is almost invariable. However, water molecules adsorbing on NZVI particle is thicker with the particle size increasing.

## Figures and Tables

**Table 1:** Distances of two NZVI particles in initial system and minimization system of systems 1–5.

Simulation system	System 1	System 2	System 3	System 4	System 5
Distance of initial system (nm)	0.7	0.85	0.94	0.111	0.126
Distance of minimization system (nm)	0.78	0.88	0.1	0.125	0.135

**Table 2:** Average aggregation speeds of systems 1–5.

Simulation system	System 1	System 2	System 3	System 4	System 5
Displacement distance (nm)	0.53	0.63	0.75	1	1.1
Aggregation time (ps)	20	32	50	84	109
Average aggregation speed (nm/ps)	0.0265	0.0197	0.015	0.0119	0.0101
